# Changes in the Levels of Stress Perception, Experiencing Depressive Symptoms and Health-Related Quality of Life of Residents after the 2016 Gyeongju Earthquake

**DOI:** 10.3390/ijerph18020540

**Published:** 2021-01-11

**Authors:** Young Ran Han, Yeo Won Jeong, Sang Kyu Kim, Han Seok Jeong

**Affiliations:** 1 Department of Nursing, College of Medicine, Dongguk University, Gyeongju 38066, Korea; hanyr@dongguk.ac.kr; 2 Department of Preventive Medicine, College of Medicine, Dongguk University, Gyeongju 38066, Korea; dunggly@dongguk.ac.kr; 3 Department of Statistics, Dongguk University, Gyeongju 38066, Korea; hsjeong@ncc.re.kr

**Keywords:** earthquakes, quality of life, depression, psychological stress

## Abstract

Background: The aim of this study was to evaluate the impact of the 2016 Gyeongju Earthquake on the stress perception, depressive symptoms, and health-related quality of life (HRQoL) among Gyeongju residents. Methods: This study was a secondary analysis of the 2015–2017 Korean Community Health Survey undertaken in the disaster area, Gyeongju, and in controlled areas, Sangju and Yangju, which had varying seismic intensities. Pearson’s chi-square test, ANCOVA and two-way ANOVA were performed. Results: The stress perception rate and anxiety/depression in the 5th dimension of the EuroQul-five-dimensions three-level version (EQ-5D-3L) in Gyeongju was significantly higher in 2017 than in 2016. As for the HRQoL, the controlled regions showed a tendency to increase in 2017 rather than in 2016, while Gyeongju had no significant differences during 2015 and 2017. As a result, Gyeongju had the lowest HRQoL in 2017. Conclusion: Mental health in the disaster area after the 2016 earthquake was worse, and the HRQoL of Gyeongju residents was relatively lower than the control regions. Based on the results of the study, government agencies should remain interested in developing a post-disaster psychological support program for disaster survivors at a community level.

## 1. Introduction

After sudden and unexpected disasters, victims experience psychological and physical health problems apart from financial and social losses. Victims’ quality of life (QOL) is also affected by the complex interaction of these factors [1,2,3,4,5]. The health-related effects of earthquakes not only include physical health problems but also psychological and mental health issues such as stress, depression, and post-traumatic stress disorder (PTSD) [6,7]. A disaster may act as a trigger that leads to an acute stress response developing into PTSD, and stress that causes changes in lifestyle patterns, such as a disaster, leads to a substantial burden on mental health [8]. This can lead to anxiety, depression, and PTSD, which are specific psychological problems following major trauma such as disasters [8,9]. Recently, studies on psychiatric problems, such as stress, depression, and anxiety after a disaster are being conducted [10,11]. However, only a few studies involved looking at how all three major psychiatric conditions—stress, anxiety, and depression—impact on victims after a disaster [10,12,13]. Additionally, studies that have investigated disasters’ comprehensive effects, such as post-disaster QOL, are limited in South Korea [5,7]. South Korea has a higher age-standardized mortality rate via suicide (24.6) than other members of the Organization for Economic Co-operation and Development (OECD), which averages 11.2 [14], and the lifetime prevalence of major depressive disorder has increased two-fold to 5.3% in 2013 from 2.8% in 2012 [15]. As psychiatric problems in South Korea increase, interest by government and researchers on the mental health issues of earthquake survivors is essential.

On 12 September 2016, a foreshock with a magnitude of 5.1 on the Richter scale occurred in the city of Gyeongju in North Gyeongsang Province, followed by a 5.8 magnitude earthquake. It was the biggest earthquake recorded since the Korea Meteorological Administration began instrumental seismic monitoring in 1978. A total of 632 aftershocks of various magnitudes were recorded until 21 August 2017. No deaths were reported in the 2016 Gyeongju Earthquake (referred to as the 2016 earthquake hereafter), but 57 people suffered minor injuries [6,7]. The province sustained property damages estimated at KRW 10.6 billion, apart from damage to cultural assets in the Daeungjeon of the Bulguksa Temple and the Cheomseongdae Observatory [6,7]. The repeated aftershocks not only increased the levels of anxiety amongst Gyeongju residents [6,16] but also may have affected their overall QOL [7] due to the subsequent difficulties in their daily lives. Since Gyeongju has many of Korea’s World Heritages sites, designated by United Nations Educational, Scientific and Cultural Organization (UNESCO), including Seokguram, Bulguksa, and Yangdong villages [17], it is an area where school trips, academic meetings, and international events are held. Therefore, residents working in tourism were also financially threatened [6,7] by the significant decline in the number of tourists.

**Figure 1:**
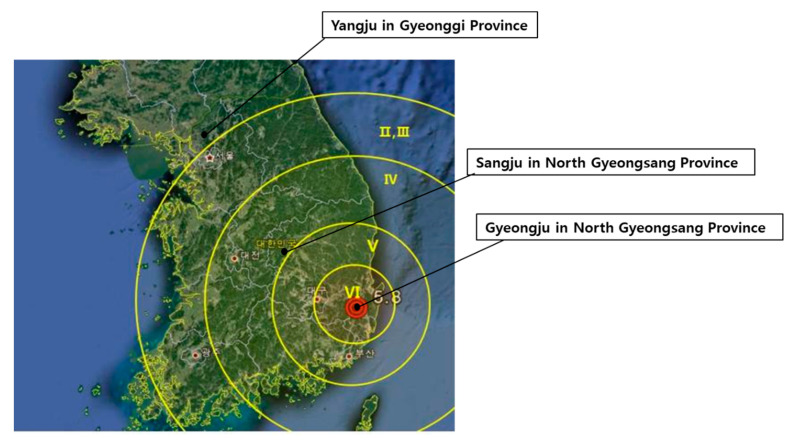
Distribution of seismic intensity.

This study aimed to evaluate the impact of the 2016 Gyeongju Earthquake on the stress perception, depressive symptoms, and health-related quality of life (HRQoL) of Gyeongju residents. We selected three regions to compare the changes in the main variables: Gyeongju as the disaster area and two control areas—Sangju and Yangju—that had different seismic intensities. Sangju is in North Gyeongsang Province, and it is far from earthquake-prone areas. Yangju is in Gyeonggi Province, and it reported the lowest seismic intensity among the three regions according to the Korea Meteorological Administration [16] (Figure 1).

Lee (2014) analyzed 50 disaster-related nursing articles published from 1995 to 2014 in Korea, and the results showed an increasing trend in the number of disaster-related nursing articles per year [18]. Research on psychological health problems, such as stress, as a direct effect of natural disasters consists of cross-sectional surveys of some survivors; however, no studies have been conducted at the community level [18]. Studies specifically on the 2016 earthquake include a qualitative study on “victims’ daily life experience” [19], a structural model research on the QOL of victims in the cities of Gyeongju and Pohang [20], and the life satisfaction of Gyeongju residents [7]. Meanwhile, international research related to earthquakes has examined the physical and psychological health as well as the QOL of specific victim groups such as college students, soldiers performing earthquake recovery work, firemen, and earthquake survivors [16,21,22,23]. In addition, there was a study on the prevalence rate of depression six months after the attack on the World Trade Center [24], and a study on PTSD after the Wenchuan earthquake at the community level [25]. Studies have also been conducted on the standardized mortality ratio from renal failure and infant and child mortality before and after the 2011 earthquake and tsunami in Japan using secondary data analysis of large population-based survey data [26,27].

Support for disaster damage has mostly been material and financial in nature. Disaster psychology, such as psychological counseling and therapy for disaster victims at the community level, has been relatively neglected. As the number of various natural and social disasters, such as typhoons, flooding, explosion, and fires, is increasing in South Korea, a long-term epidemiological survey is necessary to assess the post-disaster psychological and mental health problems and QOL, which can help authorities address these issues [2,3,4,5]. A community-based response is crucial at an early stage, as it can make a significant difference to residents and should be emphasized immediately [3].

The Community Health Survey (CHS) is a large-scale survey conducted from mid-August to the end of October every year under the supervision of the Korea Centers for Disease Control and Prevention (KCDC) [28]. It provides reliable data collected from households selected by systematic sampling from all 254 local districts in Korea [28]. Data sets surveyed at the national level are helpful for explaining phenomena or trends in the past or over time [29].

## 2. Materials and Methods

### 2.1. Study Population

CHS is an annual national cross-sectional survey based on a standardized questionnaire undertaken by the KCDC to evaluate the health status of the adult population (≥19 years). The CHS is a large-scale sample survey conducted from mid-August to the end of October every year from 2008 in compliance with Article 4 of the Regional Public Health Act (community health status survey) and its enforcement decree [30]. The CHS uses two-stage sampling: the first stage is to apply a probability proportional to the size sampling strategy (to select primary sampling units) and the second stage is to apply systematic sampling (selecting households) [28].

For evaluating the mental health problems and HRQoL of residents in Gyeongju, we selected two control areas—Sangju and Yangju. These three cities are urban and rural complex cities that have both urban and rural characteristics. This is one of the six regional types in the CHS such as special cities and metropolitan cities [31]. As of December 2016, the population of Gyeongju was 259,452, Sangju was 101,799, and Yangju was 205,513 [32]. As Gyeongju has been the capital city for approximately a thousand years in the Silla era [33], it has the largest number of the World Heritage sites in Korea. Sangju is an historical city, as it was the capital of North Gyeongsang Province in the past [34], and Yangju is a tourist city with national parks [35]. Besides the tourism industry, Gyeongju and Sangju residents engage in agriculture, livestock rearing or fishing. Yangju residents engage in secondary and tertiary industries such as manufacturing business [33,34,35]. As the urban population has grown and become concentrated with younger adults looking for a better life, the non-metropolitan and rural area’s population has decreased in Korea [36]. Therefore, Gyeonggi Province, which includes Yangju, has the highest population rate in Korea, while Gyeongsangbuk-do, where Gyeongju and Sangju are located, is a rural city with a declining population [37]. Researchers have used the raw data from these areas according to the 2015–2017 CHS [28].

The target sample population in each area was approximately 900 adult residents. The data in our analysis were from a total of 8102 Gyeongju, Sangju, and Yangju area residents including 903, 897, and 912 people in 2015; 896, 900, and 909 people in 2016; 898, 891, and 896 people in 2017, respectively. “Raw data” refer to the “public use of raw data” defined in the Korea Centers for Disease Control and Prevention Regulation on the Disclosure Procedures of Raw Data. Refined data, which are free from entry and survey errors, were used for the present study after obtaining review exemption for the use of data from the Institutional Review Board of the university with which the author is affiliated.

### 2.2. Measurements

#### 2.2.1. Stress Perception Rate

To measure the level of stress subjectively felt in daily life, we used data concerning a question in the CHS survey: “How much stress do you feel in your daily life?” The participants responded, “very much”, “much”, “not much”, or “very little”. The present study calculated “yes” as the proportion of the respondents who answered, “very much” or “much” and “no” as the proportion of “not much” or “very little”.

#### 2.2.2. Rate of Experiencing Depressive Symptoms

The rate of experiencing depressive symptoms referred to the proportion of respondents who answered “yes” to the question, “Have you ever felt sad or despair that was strong enough to interfere with your daily life for two consecutive weeks or longer during the past year?”.

#### 2.2.3. Health-Related Quality of Life (HRQoL)

HRQoL was measured using EQ-5D-3L (EuroQul-five-dimensions three-level version), developed by the EuroQol Group [38]. The EQ-5D-3L consists of a total of five dimensions: exercise ability, self-care ability, daily activities, pain/discomfort, and anxiety/depression. Each dimension is answered by three levels, and an example of the answer “anxiety/depression” is as follows: “no problem (I am not anxious or depressive)”, “some problems (I am sometimes anxious or depressive)” or “extreme problems (I am extremely anxious or depressive).” The EQ-5D-3L index was calculated using the Korean value set of the Korean version of EQ-5D-3L [39]. The range of the scores was from −0.171 to 1–1 indicated no problems in any of the five dimensions, 0 indicated death, and negative values indicated a worse health status than death [39]. The Korean version of the EQ-5D-3L’s Cronbach’s alpha was 0.751 [40].

### 2.3. Statistical Analyses

Sociodemographic characteristics and main variables were a statistical analysis using weighted value, in accordance with the raw data use guidelines [28,41]. Descriptive statistics were used to analyze the general characteristics of residents in Gyeongju, Sangju, and Yangju. Pearson’s chi-square test was used for analysis in order to evaluate the differences over three years of stress perception and experience of depressive symptoms in Gyeongju relative to those in the areas used as a control. A multiple logistic regression analysis was performed for sociodemographic variables controlled and the post-hoc analysis used the Scheffe test. Change in the HRQoL by years in each region was analyzed by ANCOVA with controlled sociodemographic data, and two-way ANOVA was conducted to verify the main effect of each region and year and the effect of interaction between regions and years. Statistical analyses were performed using SAS Version 9.4 (Copyright ©2013, SAS institute Inc., Cary, NC, USA). Values were considered statistically significant at *p* < 0.05.

## 3. Results

### 3.1. General Characteristics of the Subjects

The most frequent age of the participants in Gyeongju and Sangju was 45–64 years (38.7–40.1%, 38.4–39.1%) in all three years from 2015 to 2017, and that of the participants in the Yangju area was 19–44 years (44–46.4%) from 2015 to 2017. In terms of sex, the proportion of female participants was slightly higher than male participants in both the Gyeongju (50.2–50.6%) and Sangju areas (51.6–51.7%) in all three years from 2015 to 2017. That of female participants in the Yangju area (49.2–49.4%) was slightly lower than males from 2015 to 2017. The proportion of those married with a spouse was higher in Gyeongju (67.0–70.3%), Sangju (66.8–68.5%), and Yangju area (67.2–68.5%) in all three years. In terms of education level, the proportion of high school graduates was higher than others in both the Gyeongju (35.4–38.0%) and Yangju areas (41.8–46.3%) in all three years; however, the proportion of below elementary school graduates was higher than others in Sangju (27.9–33.0%). The highest proportion of the participants responded “average” to the subjective health status question in Gyeongju and Sangju (45.4–51.5%, 39.6–43%) in 2015–2017 (Table 1).

### 3.2. The Change in Mental Health from 2015 to 2017 in Gyeongju and the Control Regions

The stress perception rate of residents in Gyeongju significantly increased in 2017, being 26.3% compared to 22.2% in 2016 when the earthquake occurred (χ^2^ = 3.48, *p* = 0.031) (Table 2). The control regions, Sangju and Yangju, showed a tendency to decrease in the stress perception rate in 2017 rather than 2016 or 2015 (χ^2^ = 9.84, *p* < 0.001; χ^2^ = 8.37, *p* < 0.001).

In Table 3, after the earthquake in 2016, the rate of those experiencing depressive symptoms in Gyeongju in 2017 decreased significantly compared to 2015 (χ^2^ = 5.41, *p* = 0.005). This trend was the same in the control region, Sangju (χ^2^ = 11.58, *p* < 0.001).

### 3.3. The Differences in HRQoL in Gyeongju and Control Regions from 2015 to 2017

The averages of HRQoL in Gyeongju were 0.899, 0.898, and 0.895 in 2015, 2016, and 2017 respectively; however, it was not statistically significant (F = 0.18, *p* = 0.837) (Table 4). The proportion of reported problems in each dimension of the HRQoL in Gyeongju, the rate of the 4th dimension, “pain/discomfort”, and 5th dimension, “anxiety/depression” reported problems significantly increased in 2017 after the earthquake in 2016. On the contrary, in one of the control regions, Yangju, the residents reported a significant increase in the HRQoL (F = 12.26, *p* = 0.001) and a significant decrease in the proportion of the 4th and 5th dimensions in 2017 after the earthquake in 2016. 

**Figure 2:**
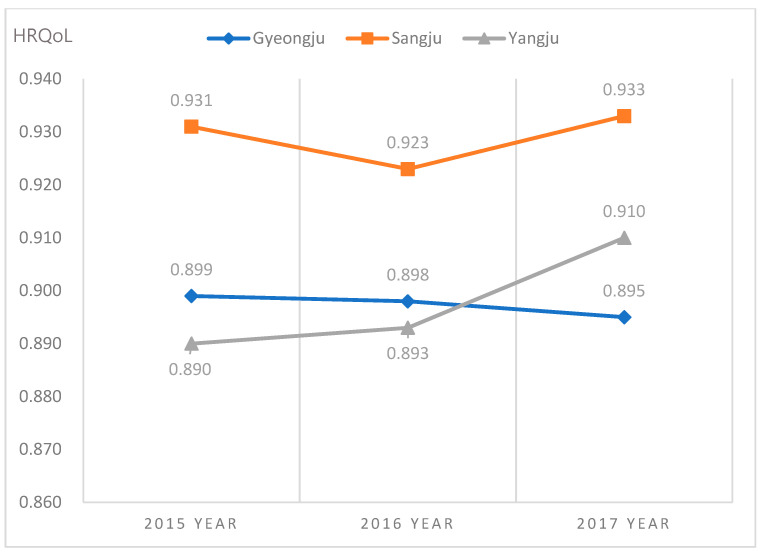
The differences in health-related quality of life (HRQoL) in regions according to year (Gyeongju = a, Sangju = b, Yangju = c; Bonferroni = a, c < b in 2015 and 2016, and a < c < b in 2017).

Two-way ANOVA was conducted to verify the main effects of each region and year on the HRQoL and the effect of interaction between region and years (Table 5). The main effect of the region on the HRQoL was significant (F = 26.07, *p* = <0.001). The interaction effect between region and year was significant (F = 4.87, *p* = 0.0006). As a result of Bonferroni’s multiple comparison of interaction effects, the HRQoL in Sangju was higher than other regions for three years. In 2017, after the earthquake in 2016, Yangju also increased significantly in HRQoL over Gyeongju (Figure 2).

## 4. Discussion

Our study analyzed changes in stress perception, depressive symptoms, and HRQoL of Gyeongju residents using the 2015–2017 CHS to identify mental health issues and change in the level of HRQoL owing to the 2016 Gyeongju Earthquake.

The results of the current study show that stress was significantly higher for residents in areas affected by the earthquake than in the control areas. In this study, the affected area, Gyeongju, reported higher rates in 2017 compared to the previous year, while the control regions, Sangju and Yangju, reported the highest stress perception rates in 2015 (Table 2). These results imply that the 2016 earthquake influenced and increased the stress perception rate of residents in the Gyeongju region in 2017. Previous studies that used different instruments from the present work have reported a significantly higher level of stress in residents in areas affected by the earthquake compared with those in unaffected areas [42] and victims having three times higher psychological stress than the others after a magnitude 6.3 earthquake [23]. Furthermore, a study showed that higher levels of exposure to a disaster, such as a tsunami, was related to more post-traumatic stress symptoms [43]. These findings of increased stress after a disaster are also consistent with our findings.

In this study, the analysis of the yearly difference revealed that Gyeongju reported a decreased rate of depressive symptoms in 2017 compared to pre-earthquake 2015 levels, and this trend was also shown in the control region, Sangju. On the other hand, the anxiety/depression dimension of the EQ-5D-3L in this study increased in Gyeongju, while the control regions, Yangju and Sangju, showed a tendency to decrease in the HRQoL, although Sangju was not statistically significant. This can be interpreted as the earthquake increased the levels of anxiety rather than depression in residents in the affected area. A previous related study on the 2008 Wenchuan Earthquake in China reported that 40.5% of participants suffered clinical symptoms of anxiety rather than depression (24.5%) [13]. Although it is not a natural disaster, the Sewol Ferry accident, a social disaster that occurred in South Korea, reported that anxiety was higher by 1.82 times than depression by 1.66 times compared with other control areas [44]. In addition, a qualitative study on the victims of the 2016 earthquake in Gyeongju reported difficulties in everyday life, such as being startled by soft sounds, inability to sleep comfortably, and being anxious and neurotic about the recurrence of earthquakes [19]. Moreover, research on PTSD and depression after hurricanes has indicated that experiences during and immediately following a disaster are the primary determinants of PTSD, with no associations between the demographic and socioeconomic characteristics of participants, whereas the risk of depression is more clearly driven by personal vulnerability such as educational level, level of family income, and number of stressors before the hurricane [45]. Age, which is a personal vulnerability, is also associated with depression after a disaster. A study showed that post-disaster, depression was more prevalent (32.8%) compared with PTSD (25.2%) in older disaster survivors [11]. Considering these findings, stress and anxiety rather than depression can assume to increase after a traumatic experience caused by earthquakes and aftershocks, whereas depression appears to have a complex mechanism that is linked to the individual’s vulnerability.

From another aspect, it can be explained, related to the onset of psychological problems after a disaster. The prevalence of psychiatric problems, such as stress, anxiety, and depression, were different after disaster and as time passed. Some studies have shown that stress or anxiety was high after disaster [13,42,43]; however, the prevalence of depression increased after a long time had elapsed, such as 2.5 or 3.3 years after the disaster occurred [10,11]. In this study, the survey was conducted 1 year after the earthquake, and it was interpreted that stress and anxiety were significantly higher than depression caused by the earthquake. In order to develop and support an appropriate psychological intervention or programs according to mental health problems as time elapses after disaster, future studies focusing on the onset and course of psychiatric problems caused by disasters are needed. In addition, it is recommended to include stress and anxiety as major variables when conducting research on the mental health of residents in the community early after a disaster.

In the case of the 2016 Gyeongju Earthquake, the following may have helped reduce the rate of depressive symptoms in 2017 as there were only minor injuries and no deaths. As the seismic center of the 2016 earthquake was deep and the duration was short, the physical damage from the earthquake was relatively minimal despite the fact that it was the biggest earthquake reported in Korean Peninsula [16,46]. In addition, the regional disaster response center provided psychological support services to victims [6]. Nonetheless, experiencing depressive symptoms by residents in Gyeongju in 2015–2017 was higher than other regions, and the proportion of problems related on the mobility and self-care in EQ-5D-3L was also higher than other regions in this study. As mentioned above, depression is not only associated with individual characteristics but also physical illness or psychosocial factors [47]; therefore, an in-depth study would be needed to identify related factors in Gyeongju.

Lastly, after the 2016 earthquake, the change in HRQoL in 2017 showed different patterns between the affected area and control areas. The level of HRQoL in the control regions showed a tendency to increase in 2017 compared to 2016, while there was no significant annual differences in the HRQoL in Gyeongju. As a result, Gyeongju had the lowest HRQoL among the three areas in 2017 and also showed a decreasing mental health dimension in HRQoL. It can be implied that the level of HRQoL in the Gyeongju area was relatively lower than the control regions after the earthquake. Our findings are consistent with those of other quality of life studies after disasters that show that the QOL of earthquake victims are significantly lower than that of people in areas not exposed to the earthquake [48], and the QOL in the psychological dimension remains poor even after three [49] to five years after the earthquake [50]. In addition, a study showed that only participants with post-traumatic symptoms among people in higher levels of exposure to disaster were related to worse qualify of life [43]. In our study, stress after the earthquake was higher than before in victim area, and it is considered to affect HRQoL in Gyeongju residents.

As South Korea is not safe from earthquakes, a comprehensive post-disaster support strategy related to psychological problems for disaster survivors, from the preparation stage to the response and recovery stages, is necessary [7,51]. Furthermore, large-scale surveys and analyses for change in psychological factors for disaster survivors, especially in affected areas, at the community or the national level are continually necessary to obtain data for psychological support programs or systems at the community level in the future. To our knowledge, the present study is the first to evaluate the mental health and the HRQoL of individuals after the Gyeongju earthquake in 2016 using reliable national data.

One of the limitations of this study was measuring “experiencing depressive symptoms” based on an affirmative or negative answer to the question. The CHS survey method is a face-to-face interview, and the possibility of not answering honestly to sensitive questions, such as depression, cannot be ruled out. However, meaningful results on mental health could be made using the dimension of EQ-5D-3L in this study. The second limitation was that we randomly chose two control regions from the many urban and rural complex cities, like Gyeongju, solely based on the seismic intensity of each region. This can be both a strength and a limitation of our research. In this study, it should be taken into account to expand the interpretation to other areas, except Sangju and Yangju, which were selected as control regions. We did not consider other factors, such as social environments or geographical features, that affected the main variables at the time of the survey. As such, it did not enable the cause–effect relationships between the earthquake and mental health including HRQoL. For this limitation, we identified trends before and after the 2016 earthquake, and we suggest that more elaborate research design, such as multilevel analysis, be required in the future.

## 5. Conclusions

We found that the stress perception rate and anxiety/depression in the EQ-5D-3L increased significantly in the affected area, Gyeongju, after the earthquake. In addition, for Gyeongju, there was no change in the average HRQoL after the earthquake; however, considering the increasing trend in the HRQoL of the control regions, it can be inferred that HRQoL of Gyeongju residents was relatively lower. The findings in this study suggest that changes in mental health problems, such as stress, anxiety, and depression, and the HRQoL for disaster survivors should be evaluated and monitored. Furthermore, it is recommended to remain interested in establishing a policy to develop and apply psychological support programs or treatment strategies in a timely manner, according to mental health problems based on the understanding of changes of stress, anxiety, and depression as time elapsed after disasters.

Considering that post-disaster depression was associated with the mortality of older disaster survivors [11], future research is needed sustainedly to examine whether post-disaster mental health problems, including depression, anxiety, and stress, are associated with increased risk of mortality among community-dwelling survivors.

## Figures and Tables

**Table 1:** Demographic characteristics of the participants (*n* = 8102).

Variables	Characteristics	Gyeongju			Sangju			Yangju		
		2015	2016	2017	2015	2016	2017	2015	2016	2017
		Total 903	896	898	897	900	891	912	909	896
		*n* (% *)	*n* (%)	*n* (%)	*n* (%)	*n* (%)	*n* (%)	*n* (%)	*n* (%)	*n* (%)
Age (years)	19–44	270 (38.8)	277 (37.8)	248 (35.6)	199 (29.7)	197 (29.1)	190 (29.5)	382 (46.4)	364 (45)	303 (44)
	45–64	376 (40.1)	265 (39.1)	339 (38.7)	344 (39.1)	361 (39.1)	347 (38.4)	332 (36.6)	344 (37.9)	345 (38.6)
	65–	25 7(21.1)	254 (23.1)	311 (25.7)	354 (31.2)	342 (31.8)	354 (32.1)	198 (17)	201 (17.1)	248 (17.4)
Sex	Male	397 (49.6)	396 (49.6)	385 (49.8)	390 (48.3)	409 (48.3)	401 (48.4)	417 (50.7)	405 (50.6)	410 (50.8)
	Female	506 (50.4)	500 (50.6)	513 (50.2)	507 (51.7)	491 (51.7)	490 (51.6)	495 (49.3)	504 (49.4)	486 (49.2)
Marital status	Married	632 (67.7)	621 (67)	649 (70.3)	598 (66.8)	632 (68.5)	611 (67.1)	639 (68.5)	613 (67.2)	621 (67.4)
	Unmarried	107 (17.5)	118 (18.1)	99 (17.4)	106 (15.4)	113 (16.9)	102 (15.6)	132 (17.9)	158 (18.9)	139 (22)
	Other	163 (14.8)	156 (14.9)	150 (12.3)	193 (17.9)	155 (14.6)	178 (17.3)	141 (13.6)	138 (13.9)	136 (10.6)
Education level	Below elementary school	48 (20.3)	219 (20.2)	250 (20.6)	358 (31.8)	310 (27.9)	354 (33)	153 (13.9)	182 (16.4)	149 (10.5)
	Middle school	113 (11.1)	98 (9.5)	100 (10)	120 (12.1)	139 (13.9)	120 (12.3)	112 (11.4)	109 (10.6)	108 (9.8)
	High school	281 (35.4)	306 (38)	282 (35.7)	233 (29.2)	259 (31.9)	244 (30.3)	365 (41.8)	373 (42.8)	381 (46.3)
	Above university	257 (33.2)	268 (32.3)	266 (33.7)	186 (26.8)	192 (26.3)	173 (24.5)	282 (32.8)	245 (30.1)	258 (33.5)
Occupations	Yes	533 (61.9)	516 (61.2)	488 (58.7)	645 (72.4)	649 (72.6)	636 (72.1)	579 (65.8)	576 (65.6)	579 (68.7)
	No	366 (38.1)	375 (38.8)	410 (41.3)	252 (27.6)	251 (27.4)	255 (27.9)	333 (34.2)	333 (34.4)	317 (31.3)
Perceived health status	Good	258 (31.8)	235 (28)	265 (33.3)	311 (37.8)	288 (36.4)	299 (36.9)	353 (41.1)	417 (46.1)	327 (40.2)
	Average	417 (47.7)	452 (51.5)	401 (45.4)	352 (39.6)	393 (43)	340 (39.6)	373 (41.2)	326 (37.1)	372 (42.7)
	Not good	224 (20.5)	204 (20.5)	232 (21.3)	234 (22.6)	219 (20.5)	252 (23.5)	186 (17.7)	166 (16.8)	197 (17.1)

Missing value excluded * weighted %.

**Table 2:** The differences in stress perception rates in the three regions from 2015 to 2017 (*n* = 8102).

	Year	Stress	2015 ^a^	2016 ^b^	2017 ^c^	χ^2^ (*p* ^2^)
Area			*n* (%) ^1^	*n* (%)	*n* (%)	Post-hoc ^3^
Gyeongju		Yes	208 (23.3)	199 (22.2)	226 (26.3)	3.48 (0.031)
		No	695 (76.7)	697 (77.8)	672 (73.7)	b < c
Sangju		Yes	184 (21.4)	151 (17.1)	116 (13.7)	9.84 (0.001)
		No	713 (78.6)	750 (82.9)	775 (86.3)	c < b < a
Yangju		Yes	307 (33.8)	244 (28.6)	238 (24.8)	8.37 (0.001)
		No	609 (66.2)	665 (71.4)	671 (75.2)	b, c < a

^1^ Weighted %; ^2^ *p*-value was calculated by chi-square tests adjusted for general characteristics (age, sex, marital status, educational level, occupations, and perceived health status); ^3^ post-hoc was used as a Scheffe test; ^a^, ^b^, and ^c^ mean the number of participants who answered ‘yes’ to the perception of stress in each region for each year, 2015 through 2017.

**Table 3:** The differences in depressive experience rate in three regions from 2015 to 2017 (*n* = 8102).

	Year	Depression	2015 ^a^	2016 ^b^	2017 ^c^	χ^2^ (*p* ^2^)
Area			*n* (%) ^1^	*n* (%)	*n* (%)	Post-hoc ^3^
Gyeongju		Yes	126 (12.9)	101 (10.3)	73 (8.1)	5.41 (0.005)
		No	777 (87.1)	795 (89.7)	825 (91.9)	c < a
Sangju		Yes	53 (4.9)	27 (2.7)	11 (1.1)	11.58 (0.001)
		No	844 (95.1)	874 (97.3)	880 (98.9)	b, c < a
Yangju		Yes	56 (5.8)	65 (7)	46 (4.7)	1.96 (0.142)
		No	860 (94.2)	844 (93)	863 (95.3)	

^1^ Weighted %; ^2^ *p*-value was calculated by chi-square tests adjusted for general characteristics (age, sex, marital status, educational level, occupations, and perceived health status); ^3^ post-hoc was used as a Scheffe test; ^a^, ^b^, and ^c^ mean the number of participants who answered ‘yes’ to the depressive experience in each region for each year, 2015 through 2017.

**Table 4:** The differences in health-related quality of life by years in each region (*n* = 8102).

EQ-5D-3L	Gyeongju				Sangju				Yangju			
	2015	2016	2017	χ^2^ or F(*p* ^2^)	2015	2016	2017	χ^2^ or F(*p*)	2015 ^a^	2016 ^b^	2017 ^c^	χ^2^ or F(*p*)
	*n* (%^1^)	*n* (%)	*n* (%)		*n* (%)	*n* (%)	*n* (%)		*n* (%)	*n* (%)	*n* (%)	
M ± SD	0.899 ± 0.00	0.898 ± 0.00	0.895 ± 0.00	0.18 (0.837)	0.931 ± 0.00	0.923 ± 0.00	0.933 ± 0.00	2.87 (0.057)	0.890 ± 0.00	0.893 ±0.00	0.910 ± 0.00	12.26 (0.001) a, b < c
Mobility												
No	710 (78.63)	705 (78.68)	708 (78.84)	2.858 (0.582)	746 (83.17)	737 (81.80)	747 (83.84)	2.850 (0.583)	785 (85.70)	762 (83.83)	784 (86.25)	8.670 (0.070)
Some	185 (20.49)	183 (20.42)	182 (20.27)		149 (16.61)	160 (17.76)	142 (15.94)		125 (13.65)	141 (15.51)	121 (123.31)	
Extreme	8 (0.89)	8 (0.89)	8 (0.89)		2 (0.22)	4 (0.44)	2 (0.22)		6 (0.66)	6 (0.66)	4 (0.44)	
Self-care												
No	813 (90.03)	833 (92.97)	827 (92.09)	6.362 (0.173)	859 (95.76)	856 (95.01)	860 (96.52)	3.714 (0.928)	868 (94.76)	850 (93.51)	872 (95.93)	12.387 (0.014)
Some	82 (9.08)	60 (6.70)	62 (6.90)		35 (3.90)	40 (4.44)	28 (3.14)		45 (4.91)	52 (5.72)	31 (3.41)	
Extreme	8 (0.89)	3 (0.33)	9 (1.00)		3 (0.33)	5 (0.55)	3 (0.34)		3 (0.33)	7 (0.77)	6 (0.66)	
Usual activities												
No	742 (82.17)	744 (83.04)	730 (81.29)	2.168 (0.704)	792 (88.29)	780 (86.57)	798 (89.56)	7.000 (0.135)	807 (88.10)	790 (86.91)	823 (90.54)	9.465 (0.050)
Some	151 (16.72)	137 (15.29)	156 (17.37)		100 (11.15)	115 (12.76)	84 (9.43)		104 (11.35)	110 (12.10)	80 (8.80)	
Extreme	10 (1.11)	15 (16.67)	12 (1.34)		5 (0.56)	6 (0.67)	9 (1.01)		5 (0.55)	9 (0.99)	6 (0.66)	
Pain or discomfort												
No	615 (68.11)	625 (69.75)	521 (58.02)	18.165 (0.001)	673 (75.03)	65 8(73.03)	629 (70.59)	6.230 (0.182)	584 (63.76)	644 (70.85)	669 (73.60)	41.472 (<0.000)
Some	251 (27.80)	237 (26.45)	342 (38.08)		216 (24.08)	223 (24.75)	245 (27.50)		298 (32.53)	228 (28.08)	231 (25.41)	
Extreme	37 (4.10)	34 (3.79)	35 (3.90)		8 (0.89)	20 (2.22)	17 (1.91)		34 (3.71)	37 (4.07)	9 (0.99)	
Anxiety or depression												
No	763 (84.50)	761 (84.93)	712 (79.29)	10.697 (0.030)	824 (91.86)	828 (91.90)	827 (92.82)	1.693 (0.792)	719 (78.49)	783 (86.14)	802 (88.23)	26.891 (<0.000)
Some	127 (14.06)	123 (13.73)	177 (19.71)		68 (7.58)	68 (7.55)	62 (6.96)		190 (20.74)	117 (12.87)	99 (10.89)	
Extreme	13 (1.44)	12 (1.34)	9 (1.00)		5 (0.56)	5 (0.55)	2 (0.22)		7 (0.76)	9 (0.99)	8 (0.88)	

^1^ Weighted %; ^2^ *p*-value was calculated by chi-square test or ANCOVA adjusted for general characteristics (age, sex, marital status, educational level, occupations, and perceived health status); ^a^, ^b^, and ^c^ mean the mean score of HRQoL in Yangju for each year, 2015 through 2017.

**Table 5:** The main effect and interaction by regions and years of HRQoL (*n* = 8102).

Variables	Sum of Squares	Degrees of Freedom	Mean Square	F	*p* ^1^
Regions	0.730	2	0.365	26.07	<0.0001
Years	0.057	2	0.028	2.02	0.1323
Regions × Years	0.273	4	0.068	4.87	0.0006
Error	113.560	8111	0.014		

^1^*p*-value was calculated by two-way ANOVA adjusted for general characteristics (age, sex, marital status, educational level, occupations, and perceived health status).

## Data Availability

Not applicable.
